# Component Mix-Match for Anatomic Total Shoulder Arthroplasty Revision: A Case Report

**DOI:** 10.7759/cureus.75092

**Published:** 2024-12-04

**Authors:** Ilias Tsolis, Fady Atia, Malin Wijeratna

**Affiliations:** 1 Hand and Upper Limb Unit, Robert Jones and Agnes Hunt Orthopaedic Hospital, Oswestry, GBR; 2 Trauma and Orthopaedics, Nottingham University Hospital, Nottingham, GBR; 3 Trauma and Orthopaedics, Northampton General Hospital, Northampton, GBR

**Keywords:** glenohumeral, mix-match, replacement, revision, shoulder

## Abstract

Although mixing and matching components is a common, safe, and well-documented practice in hip revision surgery, our extensive search indicates that it has not been previously reported for shoulder arthroplasty. This case report presents the use of mixed implants in shoulder revision surgery to reduce morbidity and address flaws in the initial implant design. We describe a case of a patient with multiple epiphyseal dysplasia who was treated for osteoarthritis in his left shoulder with an anatomic shoulder replacement in 2014. In 2018, the patient presented with deteriorating function, increasing pain, and radiological signs of glenoid component wear and loosening. A decision was made to proceed with revision shoulder arthroplasty. Given the patient's young age and intact rotator cuff function, the revision was planned as an anatomic construct. The challenge was a well-fixed, fully hydroxyapatite-coated stem and a glenoid metal-backed component with a failing polyethylene locking mechanism. After extensive discussion with the patient, a combined decision was made to retain the well-fixed humeral stem and revise the glenoid side using impaction grafting with allograft, followed by cementing an all-polyethylene glenoid from a different company. The revision surgery was performed uneventfully, resulting in pain relief and improved function beyond the levels achieved in the initial operation. Notably, the original head and the revision glenoid had a curvature radius mismatch, in contrast to the original design's absolute congruence. Recent studies suggest that such a mismatch, within limits, can recreate normal shoulder kinematics and reduce glenoid loosening.

## Introduction

Currently, available practice guidelines discourage the use of mixed implants for joint replacements. Mixing implants is against the Medical and Healthcare Products Regulatory Agency (MHRA) recommendations and directions of manufacturers [1]. Therefore, surgeons implanting unapproved mixed and matched combinations do so under their own liability [2,3]. In the UK, over 50% of revised total hip arthroplasty cases are estimated to have mixed implants [4]. This practice has been adopted to manage complex revision scenarios that would otherwise increase morbidity and is supported by positive clinical outcomes [5].

We present a case of mixing and matching components for an anatomic shoulder arthroplasty glenoid revision, which to our knowledge is the first time this has been reported in the literature.

## Case presentation

This is a case report of a 41-year-old male patient with a background history of multiple epiphyseal dysplasia. This disease is associated with early onset of osteoarthritis as well as long bone deformity [6]. By the time he sought attention for his left shoulder osteoarthritis, he had already undergone bilateral total hip replacements and a right shoulder reverse arthroplasty.

**Figure 1:**
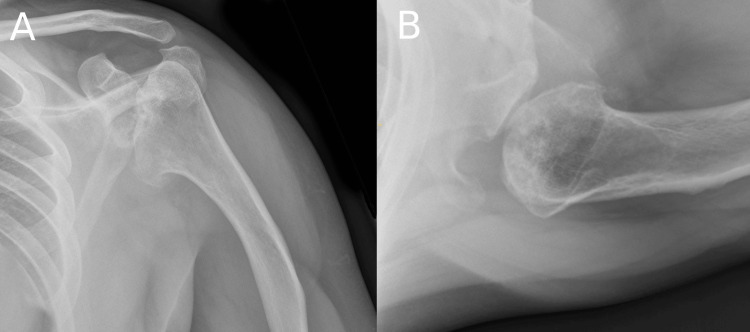
X-rays of the left shoulder at presentation (A) Anteroposterior view; (B) Axial view

**Figure 2:**
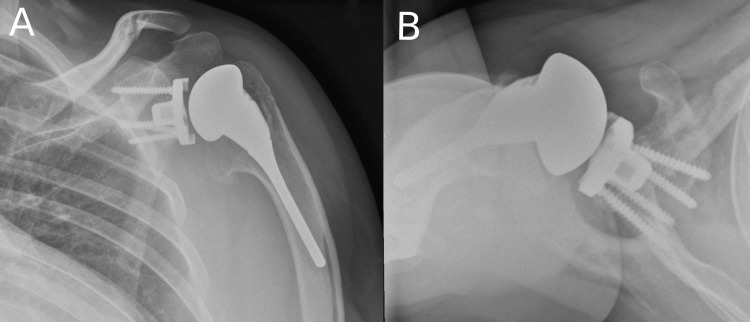
X-rays of the left shoulder immediately after the index operation. (A) Anteroposterior view; (B) Axial view

In October 2014, he presented with left shoulder pain due to glenohumeral osteoarthritis (Figure 1) and an Oxford Shoulder Score (OSS) [7] of 15 out of 48 and underwent a total shoulder arthroplasty procedure for the left shoulder in the same month (Figure 2).

In April 2015, the patient felt very satisfied with his left shoulder replacement and had returned fully to his activities of daily living and his passions, such as guitar playing. Unfortunately, this patient was lost to follow-up but was subsequently referred back to the shoulder and elbow unit via the hip clinic, where he was followed up for monitoring of his bilateral hip replacements. 

Following the re-referral, the patient was eventually seen in the shoulder clinic in October 2018 and remained satisfied with his shoulder replacements. However, during further review in November 2018, his left shoulder had deteriorated with a visual analog score (VAS) [8] of 4-5/10 and a creak felt and heard during arm elevation. The left shoulder OSS had reduced to 28/48. 

**Figure 3:**
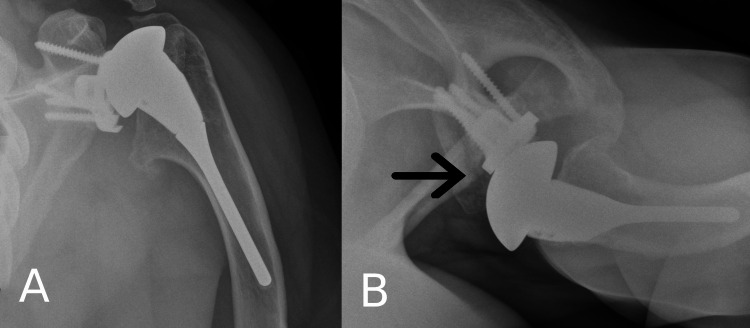
X-rays of the left shoulder revealing implant failure and anterior sublaxation (black arrow) (A) Anteroposterior view; (B) Axial view

These clinical findings were suggestive of metal-on-metal articulation, and radiographic imaging revealed early polyethylene wear with narrowing of the joint space and metal-on-metal articulation (Figure 3). His case and findings were discussed at the regional multi-disciplinary team (MDT) meeting. The initial plan was to arrange for a CT scan with metal reduction sequences for a detailed assessment of the glenoid. Three-dimensional anatomical modeling was used to aid pre-operative planning as per MDT’s recommendations. 

Following further discussion at the MDT meeting, a surgical plan was formulated to revise the patient’s shoulder replacement through a single-stage or two-stage procedure depending on the operative findings of glenoid bone stock and implant fixation. If either were compromised, the patient would require glenoid bone grafting and conversion to a hemiarthroplasty as the first part of a two-stage revision. This would be followed by a planned second stage. The option of mixing and matching components was also discussed as a possibility. Preoperative planning also included an investigation of the radius of curvature mismatches between a potential new glenoid implant while maintaining the original stem and replacing the humeral head (Table 1).

**Table 1:** Exatech-Equinoxe radial mismatch HH: humeral head Source: [9]

Radial mismatch associated with glenoid/humeral pairings (recommend shaded)						
Glenoid	38 mm HH	41 mm HH	44 mm HH	47 mm HH	50 mm HH	53 mm HH
27 mm (alpha)	7.7163	5.8647	4.2645	2.6592	1.0514	-0.5565
31 mm (beta)	11.7163	9.8647	8.2645	6.6592	5.0514	3.4435

The patient underwent a single-stage revision of his anatomic glenoid implant in August 2019. The intraoperative findings included a dissociated polyethylene bearing found in the posterior capsule with metal-on-metal articulation with posterior baseplate wear and secondary metallosis. The locking mechanism securing the polyethylene was compromised, thus preventing polyethylene exchange. However, the metal glenoid baseplate remained well-fixed, and the humeral stem was stable. This posed a technical challenge to the surgical team due to significant proximal humeral deformity in addition to the fact that attempts to explant the well-bonded, fully hydroxyapatite-coated stem would have caused significantly increased morbidity. In our experience, removing a VAIOS humeral stem (JRI Orthopaedics, Sheffield, UK) usually requires a humeral osteotomy with a window.

An intraoperative decision was made to proceed with a single-stage revision, exchanging the metal glenoid baseplate for a medium alpha-cemented Exactech - Equinoxe polyethylene implant (Exactech, Inc., Gainesville, FL, USA). The recommended matched humerus and glenoid implants had a radius of curvature that varied between 3.4 mm and 7.7 mm (Table 1). As the existing humeral head was 42 mm in diameter, the new glenoid component would result in a 5.3 mm radius of curvature mismatch. The contained glenoid bone defect was reconstructed with an impaction bone graft using a femoral head allograft. The humeral head was exchanged for a new 42 +3 VAIOS head, which was the same size and offset as the existing humeral head.

**Figure 4:**
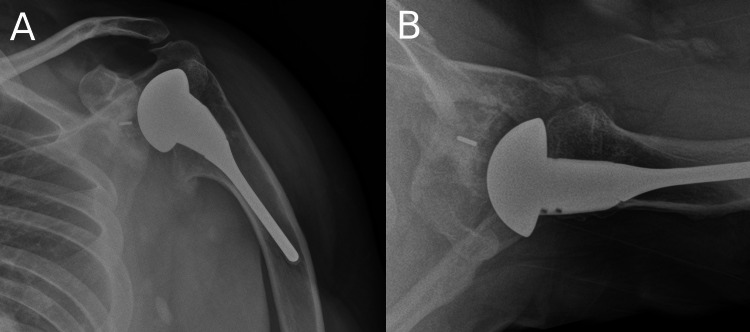
X-rays of the left shoulder displaying the revised implants at final check-up (A) Anteroposterior view; (B) Axial view

Postoperatively the patient was followed up regularly with reviews at two weeks, six weeks, 12 weeks, seven months, one year, and two years. The patient's left shoulder OSS improved to 44/48 at his two-year review. He gradually regained his range of motion with 150 degrees of forward elevation, 70 degrees of external rotation, and internal rotation to the mid-lumbar spine. He was very pleased and felt that his clinical outcome was even better than following his primary shoulder replacement. Ultimately, the patient was discharged from physiotherapy in January 2020 (Figure 4).

## Discussion

This case suggests that a positive clinical outcome can be achieved by using mixed implants in revision cases. A literature search performed in June 2024 [10] did not reveal any available published work on the use of mixed implants in shoulder arthroplasty. Currently, in the UK, the decision to mix and match implants for a revision case is guided by the work undertaken by Kelly et al. [11] on behalf of the British Shoulder and Elbow Society (BESS) for the consideration of the National Joint Registry (NJR). This piece of work is available to BESS members and advocates mixing and matching components for revision cases and not for primary shoulder arthroplasty.

In this patient, utilizing a glenoid implant from a different manufacturer enabled the surgical team to avoid a challenging two-stage revision, which would have included revising the humeral stem. This would have been fraught with difficulties and risk of significant morbidity given the need for osteotomy in an already deformed humerus. Also according to some authors, repeat shoulder operations increase the likelihood of periprosthetic infection [12].

Recent studies have suggested that radius curvature mismatch may be able to further improve clinical outcomes through approximation of natural native shoulder kinematics and reduction of glenoid loosening [13-15]. Walch et al. [16] had previously reported that optimal curvature mismatch should be above 5.5 mm and not over 10 mm, but more recent studies by Schoch et al. [15] have suggested an optimal range of 3.4-7.7 mm. Of note is that the original implants had an absolute congruency between the glenoid and the humeral head.

Our research suggests that it would be beneficial for the surgeons considering mixing and matching to calculate the radius of curvature mismatch of the planned revision implants to ensure that it is within the limits stated above, thus minimizing the chance of glenoid loosening.

## Conclusions

While mixed implants are common in total hip arthroplasty, it is unreported when mixing glenoid and humeral implants in shoulder arthroplasty. This technique is not recommended for most patients; however, it may offer a viable solution in certain cases. Specifically, mixing humeral and glenoid implants from different manufacturers might be an option when revising a well-fixed glenoid or humeral stem would otherwise lead to significant bone loss or increased morbidity. In the presented case, the patient's well-fixed, fully hydroxyapatite-coated humeral stem was retained, and the glenoid component was revised using a different company's all-polyethylene glenoid, combined with impaction grafting of the glenoid defect. This approach resulted in improved function and pain relief, illustrating that under carefully considered circumstances, such a technique can be beneficial in preserving bone stock and enhancing patient outcomes.
